# NEDD4L affects KLF5 stability through ubiquitination to control ferroptosis and radiotherapy resistance in oesophageal squamous cell carcinoma

**DOI:** 10.1111/jcmm.70062

**Published:** 2024-09-24

**Authors:** Jinjin Chen, Kaijun Ying, Jian Sun, Yao Wang, Mingming Ji, Yunhao Sun

**Affiliations:** ^1^ Department of Oncology The First People's Hospital of Yancheng City, The Yancheng Clinical College of Xuzhou Medical University Yancheng Jiangsu China; ^2^ Department of Thoracic Surgery The First People's Hospital of Yancheng City, The Yancheng Clinical College of Xuzhou Medical University Yancheng Jiangsu China

**Keywords:** DNA damage, ferroptosis, KLF5, NEDD4L, oesophageal squamous cell carcinoma, resistance

## Abstract

Oesophageal squamous cell carcinoma (ESCC) contributes to high mortality. Modulating ferroptosis may reverse resistance to radiotherapy. This article was to explore the ubiquitination modification of KLF5 and its effect on ferroptosis in ESCC. KLF5 was under‐expressed by shRNA plasmids in the cells and ROS levels were analysed by flow cytometry, ferroptotic gene expression was detected by qRT‐PCR, MDA and GSH levels were determined by ELISA, cell morphology was observed by transmission electron microscopy, and Fe ion levels were analysed by immunofluorescence. Cells were treated with Ferrostatin‐1 and NAC and analysed for cell proliferation by colony formation assay, cell migration and invasion by Transwell assays, and apoptosis by flow cytometry. DNA damage in cells was also analysed using comet assay, EdU doping assay, γH2AX fluorescence, DNA‐PKcs and PCR. NEDD4L and KLF5 binding was analysed by immunoprecipitation. Changes in ferroptosis, DNA damage and resistance were analysed in cells with both silencing NEDD4L and KLF5. Changes in tumour resistance to radiation were analysed in mice underexpressing NEDD4L and KLF5. Low expression of KLF5 significantly promotes cellular lipid peroxidation levels, with decreased expression of SOD and GPX4, and increased expression of ACSL4. Concurrently, MDA levels deplete GSH, and cells exhibit typical ferroptotic morphology with increased Fe2+ content. KLF5 inhibition results in enhanced cellular clonogenicity, migration and invasion activities, reduced apoptosis, increased tail DNA, nuclear EdU incorporation, nuclear γH2AX foci and elevated expression of DNA‐PKcs, LIG4, RAD9B and BMI1. Ferrostatin‐1 and NAC reverse these effects. NEDD4L ubiquitination modifies and degrades KLF5, with NEDD4L/KLF5 inhibition mitigating cellular ferroptosis and DNA damage, thereby promoting radiosensitivity both in vitro and in vivo. NEDD4L increases radiosensitivity by accelerating cellular ferroptosis via ubiquitination modification of KLF5.

## 1 INTRODUCTION

Oesophageal cancer ranks eighth among the cancers most frequently diagnosed and sixth among the most widely known cancer‐related cause of deaths around the world.^1^ Ninety percent of oesophageal cancers are oesophageal squamous cell carcinoma (ESCC), which contributes to high mortality due to its detection at late stages and subsequent poor prognosis.^2^ For early ESCC, an endoscopic resection is recommended, while for locally advanced ESCC, radiochemotherapy with or without resection is the standard form of therapy, and other strategies such as immunotherapy may be introduced for more challenging situations.^3^ Unfortunately, radioresistance serves as a main cause of disease progression and mortality in cancer.^4^ Evidence suggests that radiotherapy induces ferroptosis in the multiple cancers through several parallel pathways.^5^ Recent research has identified and verified a 10‐ferroptosis‐related genes signature and a nomogram that enable individualized prognosis prediction.^6^ Researchers have proposed a strategy for reversing resistance to radiotherapy by modulating ferroptosis.^7^ Hence, getting more understanding of ferroptosis and radioresistance might be conducive to the further improvement in ESCC treatment.

Neural precursor cell expressed developmentally down‐regulated 4‐like (NEDD4L), a ubiquitin protein ligase, belongs to the Nedd4 family which is capable of binding and modulating many membrane proteins to facilitate their internalization and turnover.^8^ NEDD4L plays a critical role in regulating cancer stem cells and the functions of tumour cells, proliferation, apoptosis, cell cycle regulation, migration, invasion and epithelial–mesenchymal transition, as well as tumour drug resistance.^9^ It has been noted that NEDD4L inhibits cell viability, cell cycle progression and glutamine metabolism in ESCC through the ubiquitination mechanism.^10^ Its upregulation is also recognized as a novel ferroptosis suppressor.^11^ Moreover, NEDD4L/CD71 is involved in ionizing radiation‐induced ferroptosis.^12^ Thereby, NEDD4L might act as a potential therapeutic target for ESCC.

Krüppel‐like factor 5 (KLF5) has been known to participate in cellular functions such as stemness, proliferation, apoptosis, autophagy and migration.^13^ KLF5 is an important pro‐ferroptotic regulator during the progression of acute kidney injury to chronic kidney disease and sensitizes renal epithelial cells to ferroptosis.^14^ KLF5 has been identified as a core regulatory factor in ESCC cells.^15^ By acting downstream of miR‐9, KLF5 participates in tumour growth and cancer resistance to radiotherapy + Cetuximab in vitro and in vivo.^16^ The expression and activity of KLF5 could be affected by ubiquitination.^17^ Recent researches have revealed that KLF5 promotes tumour progression and Parp inhibitor resistance in ovarian cancer.^18^ Besides, Xiaohui Shen et al demostrated that inhibits KLF5 enhanced oxaliplatin sensitivity in patient‐derived colorectal cancer organoids by restoring apoptotic response.^19^ Moreover, Juan Li et al revealed UCHL1 plays a pivotal role in TNBC by deubiquitinating and stabilizing KLF5, contributing to endocrine therapy resistance. TET1 and TET3 promote UCHL1 transcription through promoter demethylation and maintain KLF5 protein level in a UCHL1‐dependent manner, implying their potential as therapeutic targets in TNBC.^20^ Taking into consideration all above findings, we herein hypothesize that NEDD4L may interact with KLF5 through ubiquitination and thus modulate ferroptosis and radiotherapy resistance in ESCC.

## 2 MATERIALS AND METHODS

### 2.1 Cell culture and treatment

Human ESCC cell lines KYSE‐150 and TE‐1 from the Typical Culture Preservation Centre (Manassas, VA, USA) were cultured in RPMI‐1640 with 1% penicillin/streptomycin (Procell, China) and 10% FBS (ThermoFisher, VA, USA) at 37°C under 5% CO_2_. Mycoplasma contamination of cells was detected by PCR twice a month. The cells were irradiated using a cabinet irradiator (Rad Source Technologies, USA) at a dose of 6 Gy and a dose rate of 165 MU/min.

NEDD4L silencing plasmid (sh‐NEDD4L), KLF5 silencing plasmid (sh‐KLF5) and the negative control (sh‐NC) were commercially provided by GenePharma (Shanghai, China) and transfected into KYSE‐150 and TE‐1 cells using Lipofectamine RNAiMax (Life Technologies). Stable cells were selected based on antibiotic resistance at 2 μg/mL puromycin (Solarbio, Beijing, China), and samples were harvested 24 h after transfection for further study.

KYSE‐150 and TE‐1 cells were treated with 0.5 μM Ferrostatin‐1 (Fer‐1, MCE, USA), an inhibitor of ferroptosis, to rescue the cellular iron death process, and with 0.5 mM N‐acetylcysteine (NAC, MCE, USA), a free radical scavenger, to scavenge the ROS accumulation. Proteasome action was inhibited in cells using 20 μM MG132 (MCE, USA). Controls were treated with the same dose of DMSO.

### 2.2 Determination of ROS levels

ROS levels in cells were analysed by flow cytometry.^21^ Cells were incubated in 60‐mm dishes containing 5 μM BODIPY 581/591 C11 (Invitrogen, USA). After incubation at 37°C for 30 min, the cells were washed with PBS, digested with trypsin, stained with PI solution diluted in PBS for 5 min, and analysed by flow cytometry. FL1 channel signals in live cells were mapped using a CytoFLEX S instrument (Beckman Coulter, USA).

### 2.3 qRT‐PCR

TRIzol (ThermoFisher) was used to extract total RNA from ESCC cells. RNA was reverse transcribed using PrimeScript RT Master Mix (Takara, Dalian, China). The resulting cDNA was subjected to qPCR using SYBR Premix Ex Taq (Takara); then qRT‐PCR was performed on an Applied Biosystems 7500 Fast real‐time fluorescence qPCR system (ThermoFisher). The PCR products of cDNA were observed by 2% agarose gel electrophoresis. The normalized internal reference for gene expression was GAPDH, and gene expression was calculated using the 2‐ΔΔCt method. qPCR primers included: KLF5: 5′‐TGGCGTTTACGTGTGGAAGA‐3′ and 5′‐ATGTGTGTTACGCACGGTCT‐3′; superoxide dismutase (SOD): 5′‐AAAGATGGTGTGGCCGATGT‐3′ and 5′‐CAAGCCAAACGACTTCCAGC‐3′; glutathione peroxidase 4 (GPX4): 5′‐CTTTTGCCGCCTACTGAAGCC‐3′ and 5′‐CCGAACTGGTTACACGGGAA‐3′; ACSL4: 5′‐CTGGAATGACAGGCCAGTGT‐3′ and 5′‐CTGTCCCAGCACCACATGAT‐3′; LIG4: 5′‐ACTGTTGCATCTCACGTTCCT‐3′ and 5′‐AGAGTCTGTGACATCTTTGTGGT‐3′; RAD9B: 5′‐GAGGACCCATCTCGTGTGAC‐3′ and 5′‐GAGTTTGTGGCCCGATGGGTA‐3′; BMI1: 5′‐GACTCTGGGAGTGACAAGGC‐3′ and 5′‐ACTGGAGTACTGGGGCTAGG‐3′; GAPDH: 5′‐GACCCCTTCATTGACCTCAAC‐3′ and 5′‐CTTCTCCATGGTGGTGAAGA‐3′.

### 2.4 Enzyme‐linked immunosorbent assay (ELISA)

To measure malondialdehyde (MDA), cells were harvested by trypsin detachment and cell extracts were prepared by pre‐cooled lysis buffer containing thiobarbituric acid (TBA) and centrifuged at 15,000 g for 15 min at 4°C to collect the supernatant of cell lysates. The relative levels of MDA‐TBA adducts formed were indicated by the absorbance at 532 nm on a Gen5 microplate reader (BIOTEK).

Glutathione (GSH) levels were detected using the GSH‐Glo kit (Promega). Cells were harvested by trypsin detachment and added with 100 μL of prepared 1× GSH‐Glo reagent, followed by incubation for 30 min at room temperature. Next, 100 μL of complexed fluorescein assay reagent was added to each well and mixed briefly on a plate shaker. After 20 min, the cells were incubated using the Gen5 microplate reader to measure the absorbance. A standard curve of GSH concentration was generated, and the exact GSH concentration in different cells was calculated.

### 2.5 Measurement of cellular Fe2+ ion levels

Intracellular Fe2+ ion levels were measured using a Ferro‐orange kit (Dojingo, Japan). Cells were seeded on petri dishes and exposed to radiation, and added with FerroOrange (1:1000) for 30‐min incubation at 37°C. All images were acquired using a fluorescence confocal microscope (Axio Observer Z1, Zeiss, Germany).

### 2.6 Transmission electron microscopy (TEM)

Cells were collected by gently scraping and fixing the samples with 4% paraformaldehyde (PFA) (Bioss, Beijing, China) for at least 2 h at 4°C. Cells were then washed in 0.1 M sodium dimethylarsenate buffer, treated with 0.1% Millipore‐filtered dimethylarsenate‐buffered tannic acid, fixed with 1% buffered osmium, and stained with 1% Millipore filtered uranyl acetate monolithic staining. Next, the samples were dehydrated in increasing concentrations of ethanol, infiltrated, embedded, and then polymerized in a 60°C oven for approximately 3 days. Sections were cut using a Leica Ultracut sectioning machine, stained with uranyl acetate and lead citrate in a Leica EM stainer, and examined using a JEM 1010 TEM (JEOL USA, Inc.) at an accelerating voltage of 80 kV. Digital images were obtained using an AMT imaging system (Advanced Microscopy Techniques Corp).

### 2.7 Immunofluorescence

ESCC cells cultured on slides were fixed with 4% PFA for 20 min at room temperature, permeabilized with 1% Triton X‐100, closed with 5% BSA, and then incubated with primary antibody against γH2AX (ab26350, Abcam, UK) and Alexa Fluor TM 488 coupled goat anti‐mouse IgG secondary antibody (ab150113, Abcam). Cell nuclei were stained with DAPI (Beyotime, China). Fluorescence microscopy (Axio Observer Z1) was performed on slides.

### 2.8 Colony formation assay

KYSE‐150 and TE‐1 cells were cultured in 6‐well plates with 500 cells/well at 37°C under 5% CO_2_. In the second week after culture, cells were fixed with PFA and stained with 0.1% crystal violet (RIBOBIO, Guangzhou, China), and colonies containing ≥50 cells were counted.

### 2.9 Transwell assays

To examine the ability of cells to migrate and invade, Transwell assays were performed using 24‐well cellular Transwell chambers (Corning, USA) with a pore size of 8 μm. Uncoated Transwell chambers were used for migration assays, whereas Matrigel (1:6 dilution, Corning) was pre‐coated onto the upper surface of Transwell chambers in invasion assays. Cells were preconditioned by incubation in serum‐free medium overnight. RPMI 1640 medium containing 20% FBS was used as a chemotaxis inducer in the lower chamber of 24‐well plates. Single‐cell suspensions were loaded into the upper chamber. Cells were removed from the upper chamber with a cotton swab. The cells below were fixed with PFA and stained with 0.5% crystal violet before observation with a microscope (Olympus, Japan).

### 2.10 Apoptosis detection

Apoptosis was analysed using FITC Annexin V Apoptosis Detection Kit I (BD, USA). Cells were added with trypsin and resuspended, followed by incubation in 50 μL of FITC Annexin V for 10 min and in 50 μL of binding buffer for PI for 5 min at room temperature. Afterward, the cells were detected by the CytoFLEX S instrument (Beckman Coulter, USA) and Flowjo software (v10.0, Tree Star Inc, USA) to analyse the Annexin V and PI double positive cells as apoptotic cells.

### 2.11 Comet assay for DNA strand breaks

Cell suspensions were prepared in PBS and mixed with 10× volume of 0.5% low melting point agarose (Invitrogen), and then immediately pipetted onto slides pre‐treated with 1% agarose at normal melting point and cured for 10 min at 4°C. Slides were immersed in pre‐cooled alkaline lysis buffer (pH = 10) at 4°C and then placed in an electrophoresis tank containing ice‐cold electrophoresis buffer (pH >13) for 1 h to deconvolve the DNA. The slides were then washed and stained with SYBR Green I solution (Solarbio), and the cells were observed and photographed under the fluorescence microscope. A total of 100 cells in each sample were analysed by CASP software (v1.1.2, Krzysztof Końca, Poland), and the percentage of tailed DNA was used as a parameter to assess DNA strand breaks.

### 2.12 EdU assay

Cell proliferation and DNA damage repair were examined by doping with 5‐ethynyl‐2′‐deoxyuridine (EdU, Solarbio). Cells were incubated with EdU (20 μM) for 2 h at 37°C, washed and fixed with 4% PFA for 30 min, permeabilized with 0.5% Triton X‐100, and then incubated with a reaction buffer for 30 min. Cell nuclei were stained with Hoechst 33342 (Solarbio) for 5 min and then observed and photographed by fluorescence microscopy. The proliferation rate was the ratio of the number of nuclei with EdU fully incorporated to the total number of nuclei visualized by Hoechst 33342.

### 2.13 DNA‐PKcs assay

Harvested cells were fixed with 4% PFA for 15 min at room temperature, washed with PBS, and permeabilized with 90% ethanol on ice for 10 min. Cells were then washed twice with cold PBS, resuspended and incubated with primary antibody against DNA‐PKcs (#38168, Cell Signalling Tech, USA) for 1 h in PBS containing 0.5% FBS, then incubated with Alexa Fluor TM 488 coupled goat anti‐mouse IgG secondary antibody (ab150113, Abcam, UK) for 30 min in the dark, washed twice with PBS, and resuspended in 300 μL PBS. Data were analysed using a CytoFLEX S instrument (Beckman Coulter) and Flowjo software.

### 2.14 Immunoprecipitation and ubiquitination assays

Cells were harvested either by centrifugation at 300 × g for 5 min, followed by washing with ice‐cold PBS to remove residual medium. The resulting cell pellet was resuspended in 500 μL of ice‐cold lysis buffer, supplemented with protease and phosphatase inhibitors, and incubated on ice for 30 min with occasional vortexing. Lysates were then clarified by centrifugation at 14,000 × g for 15 min at 4°C, and protein concentrations were determined using a BCA protein assay. To minimize non‐specific binding, the lysates were pre‐cleared by incubating with 20 μL of Protein A/G beads for 30 min at 4°C with continuous rotation. After centrifugation at 3000 × *g* for 5 min at 4°C, the supernatant was transferred to a fresh tube. For immunoprecipitation, 500 μg of pre‐cleared protein lysate was incubated with 5 μg of primary antibody (anti‐NEDD4L (sc‐390628, Santa Cruz, Santa Cruz, USA), anti‐KLF5 (CF811860, OriGene, USA) or control IgG (sc‐2027; Santa Cruz)) overnight at 4°C on a rotator, followed by incubation with 30 μL of Protein A/G beads for 4 h at 4°C with gentle rotation. The bead‐antibody‐protein complexes were then pelleted by centrifugation at 3000 × *g* for 5 min at 4°C or by magnetic separation, and the supernatant was carefully discarded. The beads were washed three times with 1 mL of ice‐cold wash buffer, each wash followed by centrifugation at 3000 × *g* for 5 min at 4°C. The washed bead pellet was resuspended in 40 μL of 2X SDS sample buffer and incubated at 95°C for 5 min. After centrifugation at 12,000 × *g* for 5 min, the supernatant containing the immunoprecipitated proteins was collected. Finally, 20 μL of the eluted samples were subjected to SDS‐PAGE on a 10% gel, and proteins were transferred to PVDF membranes using a wet transfer system. Membranes were blocked in 5% non‐fat milk in TBS‐T for 1 h at room temperature, then incubated with primary antibodies specific to the target protein. After sufficient washing, the immune complexes were eluted, and the ubiquitination level was detected by Western blotting using an anti‐ubiquitin antibody (ab134953, Abcam).

### 2.15 Western blotting

Harvested cells were lysed in radioimmunoprecipitation assay buffer supplemented with protease inhibitors, and protein concentrations from the supernatant were determined using the BCA method. After electrophoresis, proteins were transferred onto nitrocellulose membranes (BD Pharmingen, USA), incubated with primary antibodies against NEDD4 (MAB6218, R&D Systems, USA), NEDD4L (sc‐390628, Santa Cruz), KLF5 (CF811860, OriGene, USA) and β‐actin (ab8226, Abcam) for 16 h at 4°C, followed by incubation with horseradish peroxidase‐coupled secondary antibodies (ab205719, Abcam) for 1 h at room temperature. Membranes were exposed using an enhanced chemiluminescence kit (Pierce, Rockford, IL, USA). Grey‐scale images were captured and quantified using a Gel EDAS 293 analysis system (Cold Spring USA Corporation) and Gel‐Pro Analyzer 3.1 software (Media Cybernetics), with β‐actin as an internal control.

### 2.16 Xenograft tumour model

Thirty‐six BALB/c thymus‐less (NU / NU) female mice purchased from Vital River lived in a 12/12 circadian rhythm with normal food and water. And 2 × 106 KYSE‐150 and TE‐1 cells were resuspended in 200 μL of PBS and injected into the right coeliac subgastrium of the mice. The tumours were treated with radiotherapy (Philips deep X‐ray therapy machine) at 0.44 Gy/min, 2.0 Gy at a time for 7 consecutive days. The change in tumour volume was measured every 7 days, and the volume was calculated as length × width2 /2. After 28 days, the mice were euthanized by intraperitoneal injection of 1% sodium pentobarbital (150 mg/kg), and the tumours were excised and measured for tumour weight. All procedures performed in this study involving animal models were in accordance with the ethical standards of The First People's Hospital of Yancheng City (Permit number: XMLL‐2022‐849).

### 2.17 Statistical analyses

SPSS 22.0 (IBM, USA) was used for statistical analysis. Measurement data were exhibited as mean ± SD. The unpaired *t*‐test was used for pairwise comparisons, and one‐way or two‐way ANOVA was employed for multi‐group comparisons, followed by Tukey's for post‐hoc test and log rank test for post‐statistical analyses, with *p* < 0.05 indicating statistically significant differences.

## 3 RESULTS

### 3.1 Inhibition of KLF5 promotes cellular ferroptosis

**FIGURE 1:**
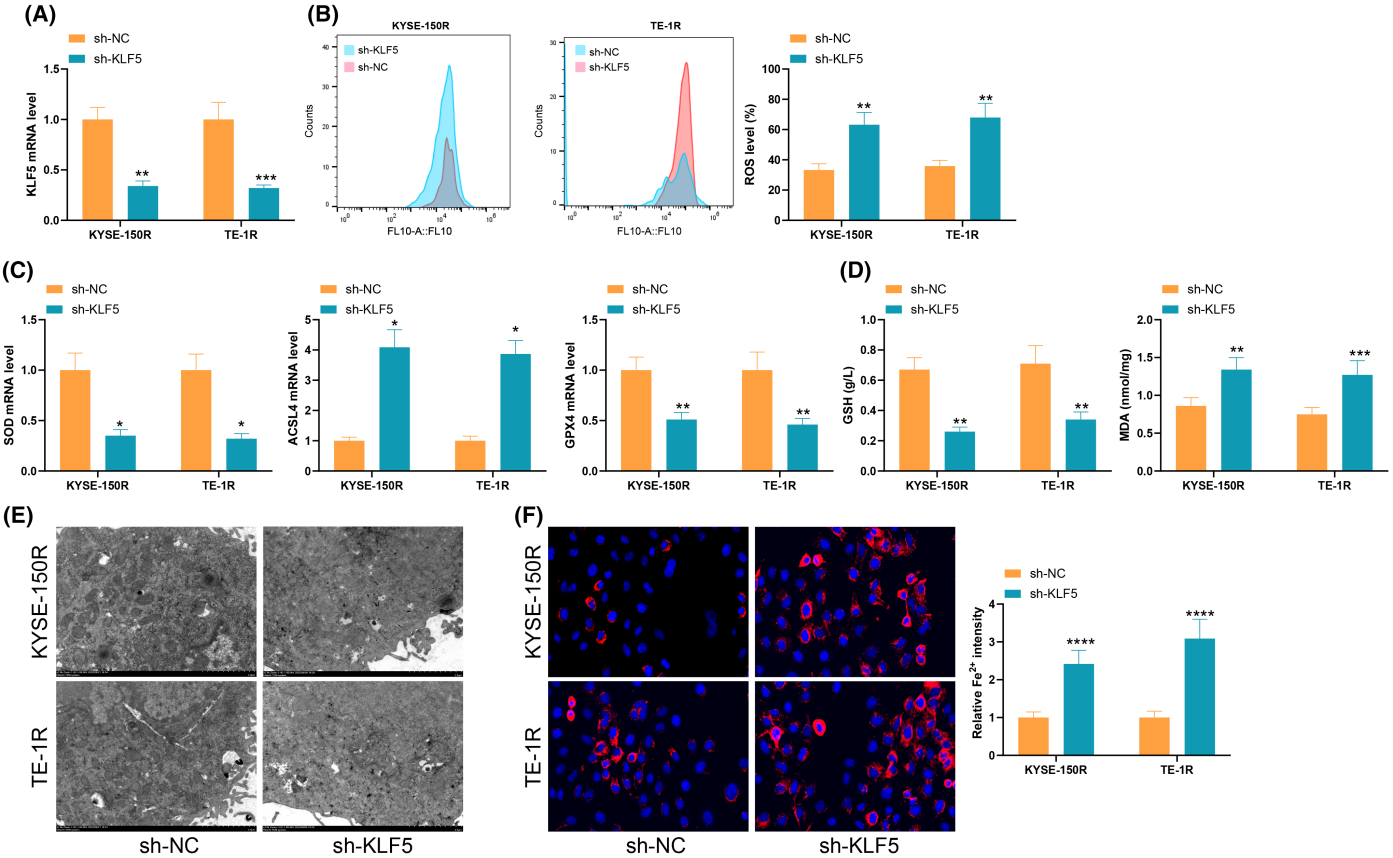
Effect of KLF5 downregulation on cellular ferroptosis. (A) qRT‐PCR to detect KLF5 expression in cells; (B) C11‐BODIPY staining to assess ROS levels in cells; (C) qRT‐PCR to detect the expression of SOD, GPX4 and ACSL4 in ESCC cells; (D) ELISA to analyse the accumulation of MDA and GSH in cells; (E) transmission electron microscopy to analyse cellular morphological changes; (F) Fluorescence intensity assessment of Fe^2+^ content in cells; Experiments were repeated three times, and data are presented as mean ± standard deviation. Statistical analysis was performed using one‐way ANOVA or 2‐Way ANOVA, followed by Tukey's post‐hoc validation, *p < 0.05, **p < 0.01, ***p < 0.001.

In the previous study, we found that overexpression of KLF5 promotes cellular radioresistance, and we inferred whether KLF5 promotes radioresistance by inhibiting ferroptosis. We constructed KLF5 low expression in KYSE‐150R and TE‐1R cells (Figure 1A). KLF5 regulates ESCC radioresistance through the Nrf2 pathway, which is an important regulator of cellular ROS levels. The results showed that KLF5 knockdown significantly promoted 6Gy radiation‐induced lipid peroxidation levels (Figure 1B); meanwhile, the levels of SOD and GPX4 were significantly reduced after KLF5 knockdown, whereas ACSL4 was significantly elevated (Figure 1C). KLF5 inhibition resulted in MDA upregulation and GSH depletion (Figure 1D). Under TEM, KLF5 low‐expressing cells showed mitochondrial atrophy, increased membrane density and rupture of the outer mitochondrial membrane, which were notable ferroptosis‐associated morphological features (Figure 1E); and assessment of the intracellular levels of Fe^2+^ using a specific fluorescent probe FerroOrange showed an increase in fluorescence intensity in KLF5 low‐expressing cells (Figure 1F). These experimental results confirmed that KLF5 inhibition led to accelerated ROS accumulation and ferroptosis in cells induced by radiotherapy.

### 3.2 sh‐KLF5 promotes radiosensitivity by inhibiting ferroptosis and ROS

**FIGURE 2:**
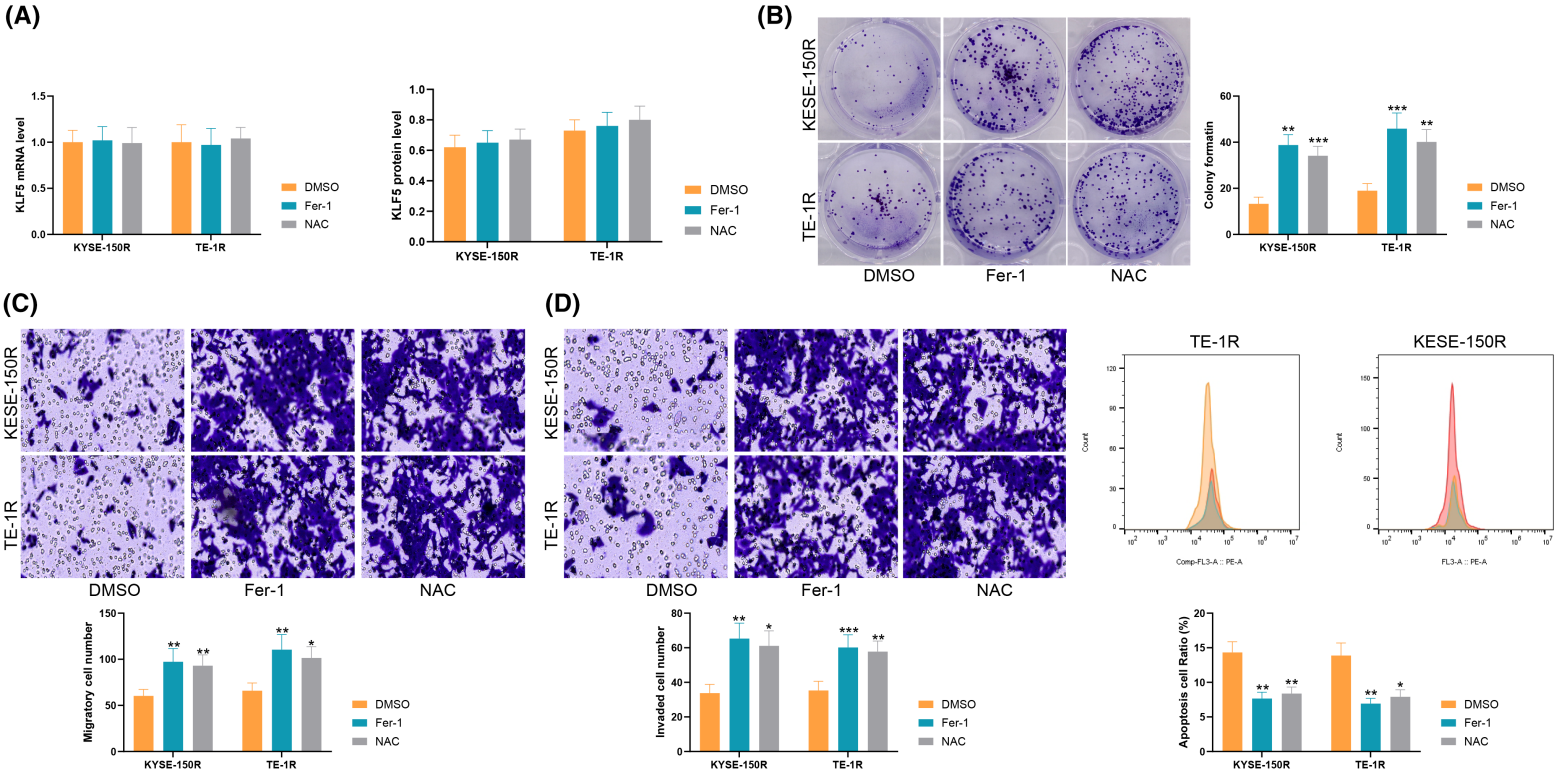
Effect of ferroptosis and ROS metabolism on cell resistance. (A) qRT‐PCR to detect KLF5 expression in cells; (B) colony formation assay to analyse cell proliferation; (C, D) Transwell assays to analyse the cell migration and invasion; (E) flow cytometry to analyse cell apoptosis; Experiments were repeated three times, and data are presented as mean ± standard deviation. Statistical analysis was performed using one‐way ANOVA or 2‐Way ANOVA, followed by Tukey's post‐hoc validation, *p < 0.05, **p < 0.01, ***p < 0.001.

Subsequently, the ferroptosis inhibitor Ferrostatin‐1 was used to treat sh‐KLF5 cells, while the free‐radical scavenger N‐acetylcysteine was used to reduce the metabolic process of ROS in the cells. Although KLF5 expression in ESCC cells did not change significantly at this time (Figure 2A), both Ferrostatin‐1 and NAC treatments resulted in elevated clonal number of cells under radiation and enhanced cell proliferative activity (Figure 2B); as well as significant increases in migrating cells (Figure 2C) and invasion ability (Figure 2D). Ferrostatin‐1 treatment significantly reduced apoptosis (Figure 2E). In short, the reversal of ferroptosis and ROS metabolism downstream of KLF5 led to the enhancement of cellular radiosensitivity.

### 3.3 KLF5 inhibition promotes cellular DNA damage

**FIGURE 3:**
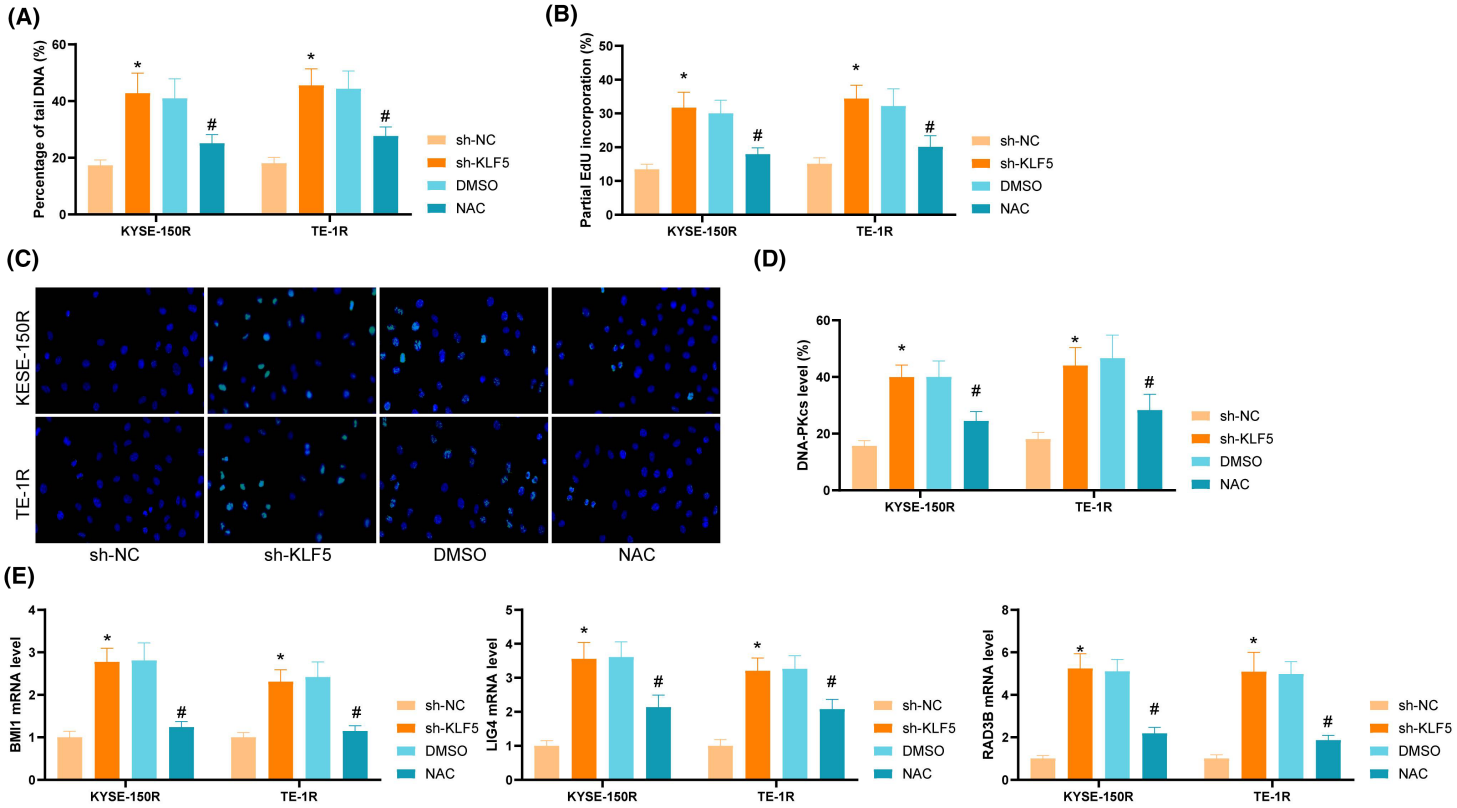
KLF5 inhibition promotes cellular DNA damage. (A) Alkaline comet assay to analyse DNA damage repair in cells; (B) EdU doping assay to analyse cellular damage; (C) Immunofluorescence to analyse γH2AX content in nuclei; (D) Flow cytometry to analyse the expression of DNA‐PKcs in cells; (E) qRT‐PCR to analyse LIG4, RAD9B and BMI1 expression in ESCC cells; Experiments were repeated three times, and data are presented as mean ± standard deviation. Statistical analysis was performed using one‐way ANOVA or 2‐Way ANOVA, followed by Tukey's post‐hoc validation, * denotes sh‐NC compared with sh‐KLF5, # denotes DMSO compared with NAC.

We further analysed whether KLF5 inhibition resulted in ROS‐dependent DNA damage. Assays in cells showed that KLF5 inhibition induced the production of significant single strand breaks (SSBs) and double‐strand breaks (DSBs) with increased percentage of tail DNA. ROS scavenger NAC reduced the partial DNA percentage (Figure 3A). DNA damage in cells was also confirmed by EdU assay, whereas NAC incubation resulted in a decrease in the percentage of EdU (Figure 3B). Early DNA damage and homologous recombination marker of DNA DSB repair pathway activation γH2AX were detected. The results exhibited that KLF5 inhibition resulted in greater recruitment of γH2AX to the nucleus, while NAC inhibited the nuclear translocation of γH2AX (Figure 3C). DNA‐PKcs, another protein that mediates the non‐homologous end‐joining DNA DSB repair pathway, was significantly increased after KLF5 inhibition, and partially decreased after NAC treatment (Figure 3D). The levels of several genes involved in the DNA repair pathway, LIG4, RAD9B and BMI1, were elevated upon KLF5 inhibition to different degrees in ESCC cells, and NAC treatment rescued the negative effects of KLF5 inhibition on these genes (Figure 3E). Altogether, DNA damage was aggravated by KLF5 inhibition and NAC treatment reduced DNA damage.

### 3.4 NEDD4L accelerate KLF5 protein instability via ubiquitination

**FIGURE 4:**
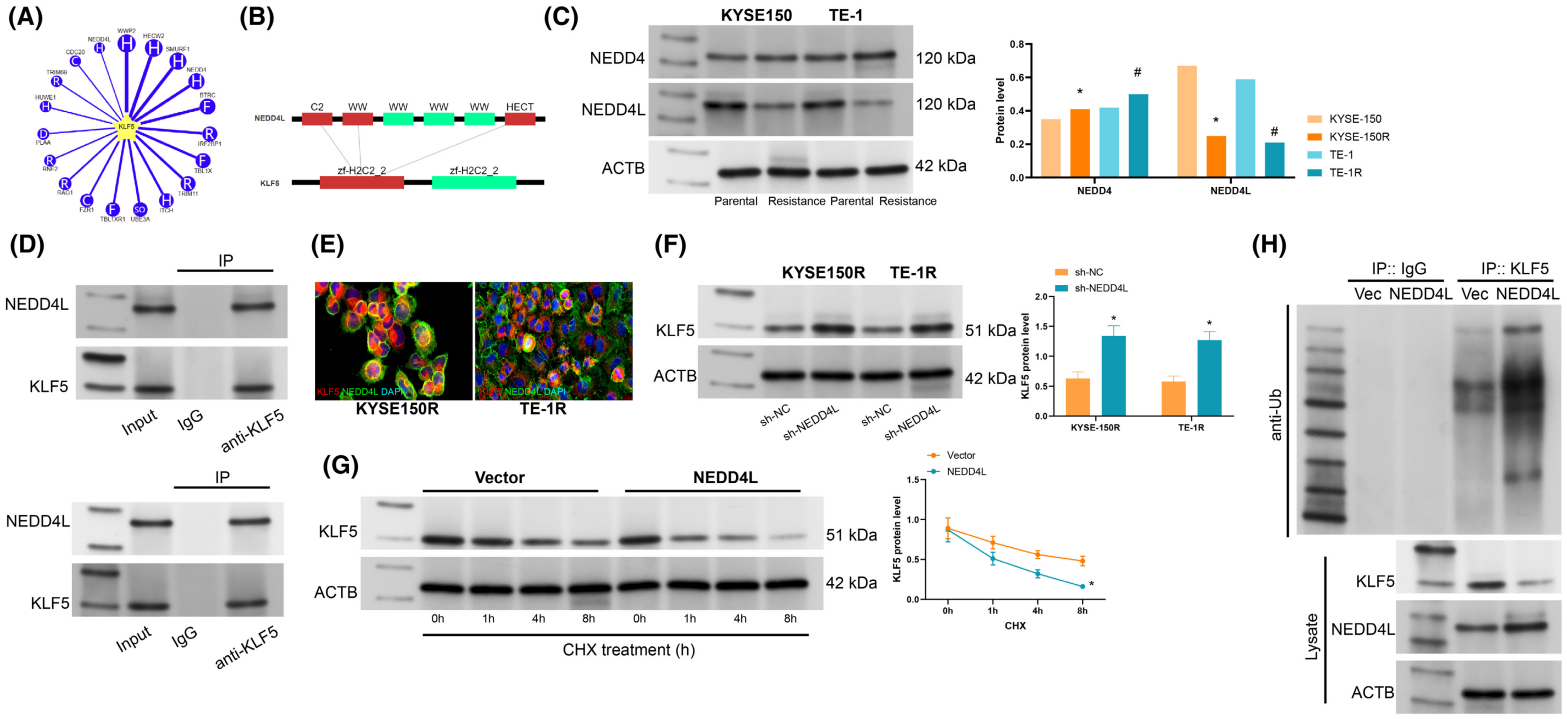
NEDD4L accelerate KLF5 protein instability via ubiquitination. (A) Ubibrowser (http://ubibrowser.bio‐it.cn/ubibrowser_v3/) analysis of the ubiquitination ligase of KLF5; (B) analysis of the structural domains linking NEDD4L to KLF5; (C) Western blotting to detect the levels of NEDD4 and NEDD4L proteins in ESCC‐resistant cells; (D, E) Immunoprecipitation and co‐immunofluorescence staining analysis of NEDD4L and KLF5 interaction; (F) Western blotting to detect KLF5 protein level after NEDD4L downregulation; (G) Western blotting to detect KLF5 stability after CHX treatment; (H) Immunoprecipitation analysis of KLF5 ubiquitination in cells; Experiments were repeated three times, and data are presented as mean ± standard deviation. Statistical analysis was performed using one‐way ANOVA or 2‐Way ANOVA, followed by Tukey's post‐hoc validation, * indicates KYSE‐150 compared with KYSE‐150R, # indicates TE‐1 compared with TE‐1R.E ~ F, *p < 0.05 according to two‐way ANOVA.

To investigate the modification of KLF5 at the protein level, the possible E3 ligases of KLF5 were analysed. E3 ligases NEDD4 and NEDD4L both modified KLF5 by ubiquitination (Figure 4A). They both acted on the zf‐H2C2_2 domain of the substrate KLF5 through the C2, WW and HECT structural domains (Figure 4B). Analysis of the ubiquitinase protein content in cells showed that NEDD4L was significantly reduced in ESCC‐resistant cells (Figure 4C). NEDD4L interacted with KLF5 in the cells via Co‐IP and immunofluorescence staining (Figure 4D,E). Subsequently, we under‐expressed NEDD4L in ESCC cells and found that the reduction of NEDD4L protein level was accompanied by a significant increase in KLF5 protein level (Figure 4F). To further determine whether NEDD4L inhibited KLF5 stability, ESCC cells were treated with CHX, and the half‐life of KLF5 was measured. It was found that NEDD4L resulted in a significant reduction in KLF5 stability (Figure 4G). NEDD4L induced c‐Myc ubiquitination to reduce its protein level in ESCC cells (Figure 4H). These findings suggest that NEDD4L interacts with KLF5 and induces ubiquitination to destabilize KLF5.

### 3.5 NEDD4L promotes KLF5 degradation and induced cellular ferroptosis

**FIGURE 5:**
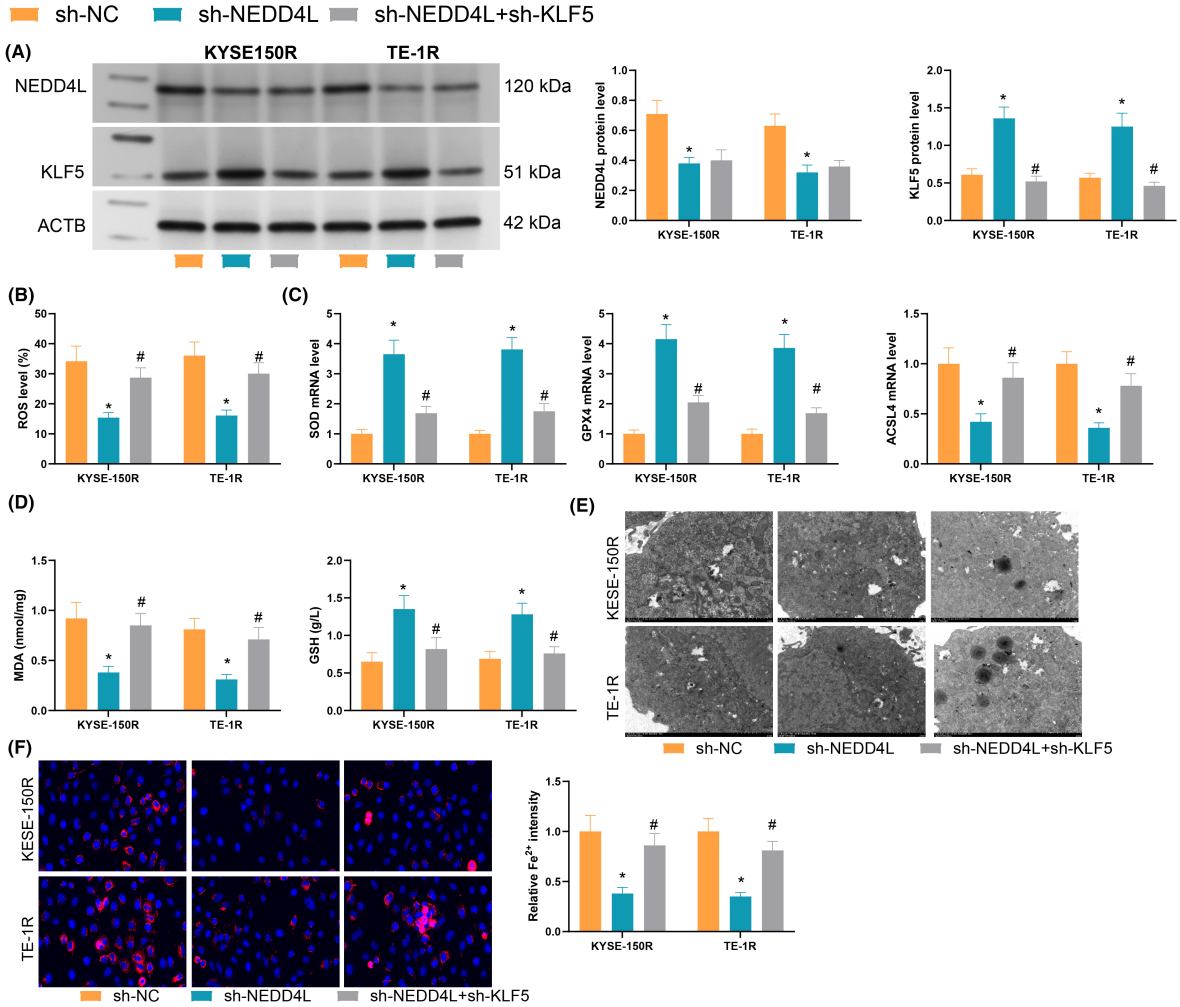
NEDD4L promotes KLF5 degradation and induced cellular ferroptosis. (A) Western blotting to detect protein levels of NEDD4L and KLF5 in cells; (B) Flow cytometry to detect ROS content in cells; (C) qRT‐PCR to detect ferroptosis gene expression in cells; (D) ELISA to detect MDA and GSH content in cells; (E) Transmission electron microscopy observation of ferroptosis morphology; (F) Immunofluorescence to detect Fe2+ content in cells; Experiments were repeated three times, and data are presented as mean ± standard deviation. Statistical analysis was performed using one‐way ANOVA or 2‐Way ANOVA, followed by Tukey's post‐hoc validation, * denotes comparison of sh‐NC with sh‐NEDD4L, # denotes comparison of sh‐NEDD4L with sh‐NEDD4L + sh‐KLF5.

NEDD4L and KLF5 were silenced in cells by shRNAs targeting NEDD4L and KLF5 (Figure 5A). NEDD4L downregulation resulted in increased ROS accumulation in cells, and KLF5 downregulation reversed this effect and elevated cellular ROS levels (Figure 5B). Inhibition of NEDD4L concurrently suppressed ferroptosis marker gene ACSL4 expression and promoted SOD and GPX4 contents. sh‐KLF5 conversely resulted in the high expression of ACSL4 and low expression of SOD and GPX4 (Figure 5C). Cellular MDA content was reduced by sh‐NEDD4L, while GSH accumulation was increased, but these effects were reversed by sh‐KLF5 (Figure 5D). The cellular ferroptosis morphology feature became inconspicuous after NEDD4L downregulation, and KLF5 inhibition accelerated the generation of cellular ferroptosis morphology (Figure 5E); the fluorescence intensity of Fe2+ was reduced after NEDD4L inhibition and then increased again after further KLF5 inhibition (Figure 5F). These data verified the role of NEDD4L/KLF5 axis on ferroptosis.

### 3.6 Knocking down NEDD4L inhibits cellular DNA damage in shKLF5 cells

**FIGURE 6:**
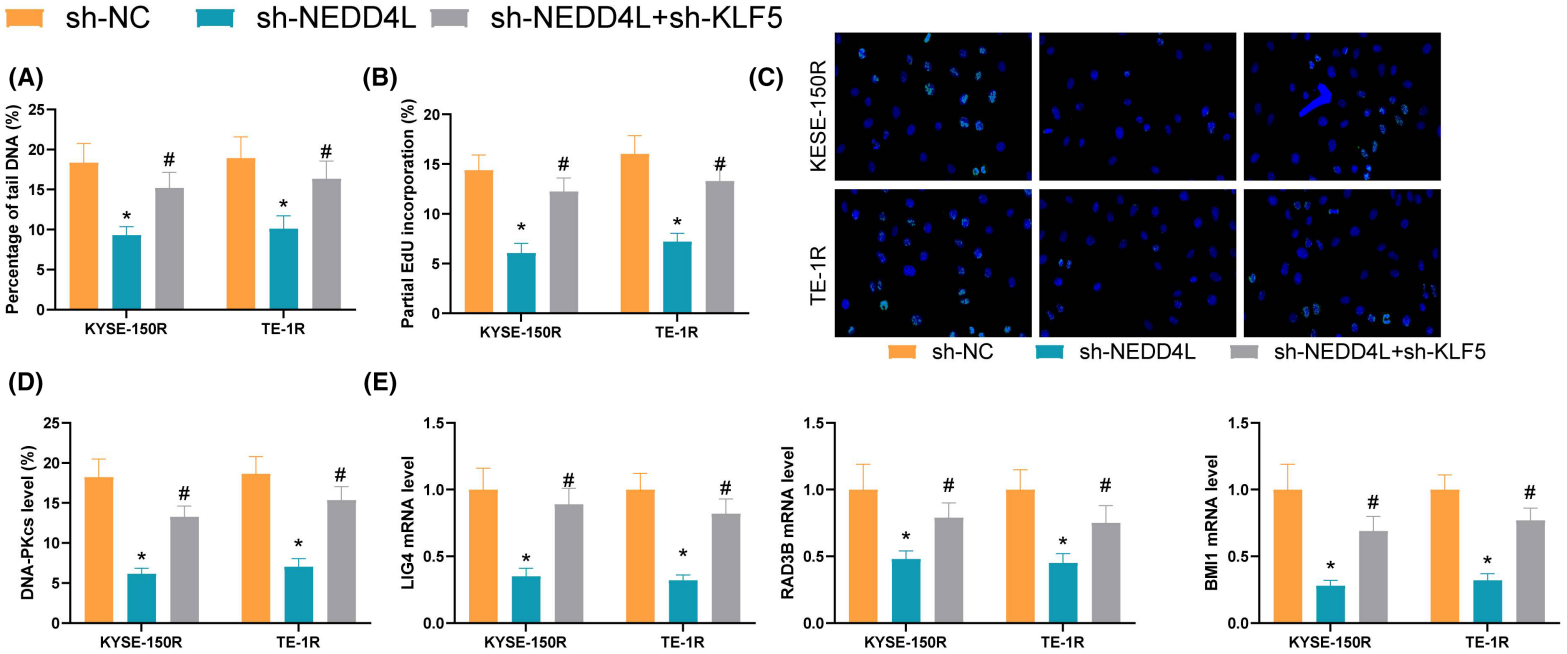
Knocking down NEDD4L inhibits cellular DNA damage in shKLF5 cells. (A) Comet assay to analyse the tail DNA content in cells; (B) EdU doping to detect DNA damage in cells; (C) Immunofluorescence to analyse γH2AX content in the nucleus; (D) Flow cytometry to analyse the content of DNA‐PKcs‐positive cells; (E) qRT‐PCR to detect the DNA damage‐related gene expression; Experiments were repeated three times, and data are presented as mean ± standard deviation. Statistical analysis was performed using one‐way ANOVA or 2‐Way ANOVA, followed by Tukey's post‐hoc validation, * denotes sh‐NC compared with sh‐NEDD4L, # denotes sh‐NEDD4L compared with sh‐NEDD4L + sh‐KLF5.

We then examined DNA damage in cells. Silencing NEDD4L decreased the amount of tail DNA in cells, and KLF5 inhibition increased the percentage of tail DNA (Figure 6A). EdU was significantly reduced after NEDD4L inhibition and increased after KLF5 inhibition (Figure 6B); γH2AX recruited in the nucleus was reduced after NEDD4L silencing, while its content in the nucleus was significantly increased after inhibition of KLF5 (Figure 6C). DNA‐PKcs‐positive cells also significantly reduced after NEDD4L inhibition and increased after KLF5 inhibition (Figure 6D). LIG4, RAD9B and BMI1 were all suppressed after NEDD4L inhibition, and increased after KLF5 silencing (Figure 6E). Collectively, downregulation of NEDD4L reduced the DNA damage repair, and KLF5 as the downstream of NEDD4L reversed the effect of NEDD4L downregulation on ESCC cells.

### 3.7 NEDD4L‐mediated KLF5 degradation promotes cellular radiosensitivity

**FIGURE 7:**
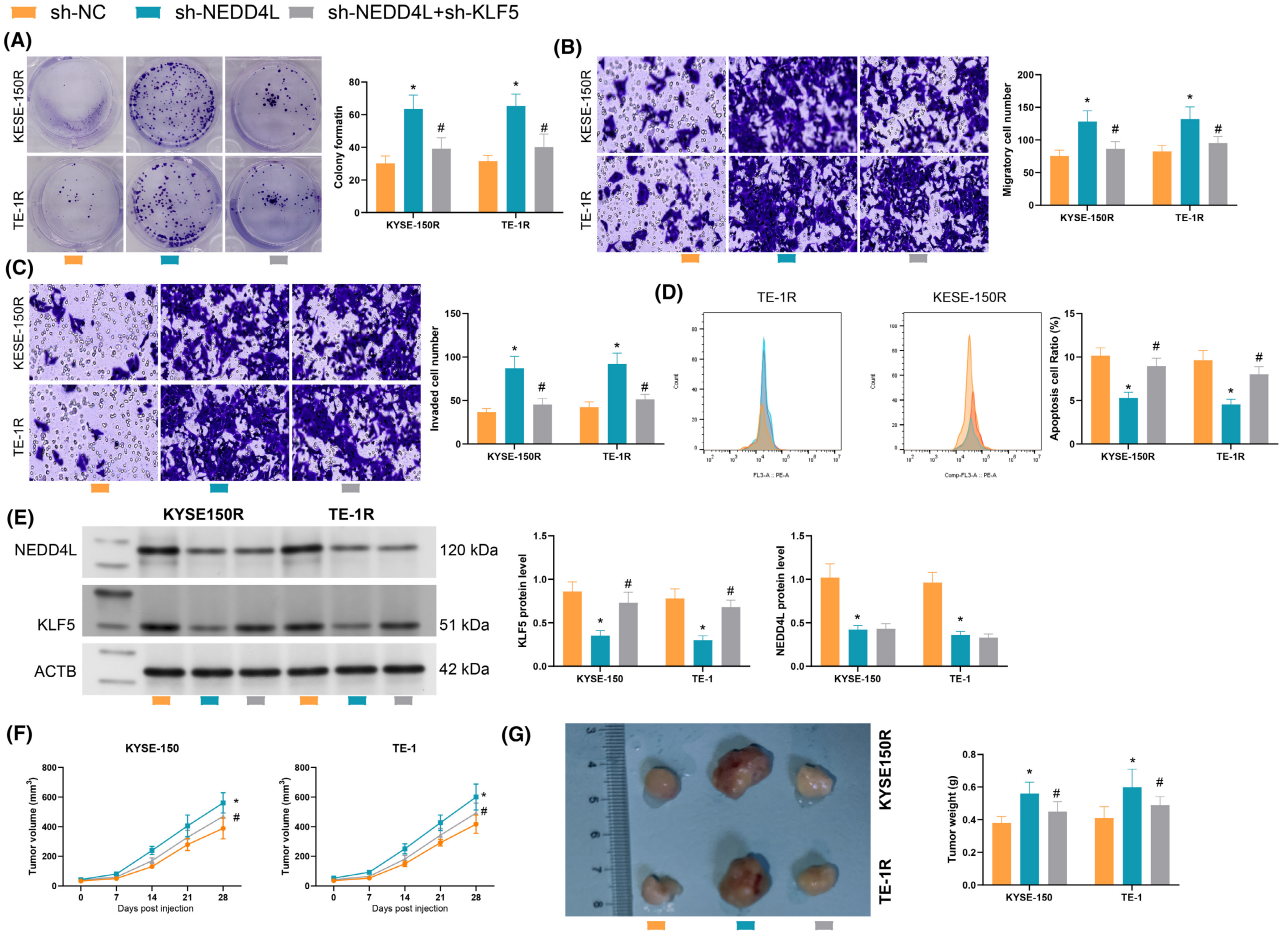
NEDD4L‐mediated KLF5 degradation promotes cellular radiosensitivity. (A) colony formation assay to detect cell proliferation; (B, C) Transwell assays to analyse the cell migration and invasion; (D) flow cytometry to detect cell apoptosis; (E) Western blotting to detect NEDD4L and KLF5 protein levels in mice; (F) Tumour volume growth curve to analyse tumorigenic activity; (G) Tumour weight to analyse cell tumorigenic ability; Experiments were repeated three times, and data are presented as mean ± standard deviation. Statistical analysis was performed using one‐way ANOVA or 2‐Way ANOVA, followed by Tukey's post‐hoc validation, * denotes the comparison of sh‐NC with sh‐NEDD4L, # denotes the comparison of sh‐NEDD4L with sh‐NEDD4L + sh‐KLF5.

After radiation treatment, downregulation of NEDD4L elevated the clonal proliferation, while KLF5 inhibited colony formation (Figure 7A); the number of migrating cells with low expression of NEDD4L was increased, and further KLF5 inhibition resulted in the diminished ability of the cells to migrate (Figure 7B); the trend of invasion assay was the same as that of the migration assay. KLF5 inhibition rescued the inhibitory effect of NEDD4L inhibition on cell invasion (Figure 7C). Apoptotic activity was significantly decreased after NEDD4L downregulation and increased after KLF5 downregulation (Figure 7D). Tumour formation under irradiation in vivo was simulated after the sh‐NEDD4L and sh‐KLF5 were delivered to the mice (Figure 7E), showing that NEDD4L inhibition accelerated the rate of ESCC tumour generation, while KLF5 inhibition conversely suppressed tumour growth (Figure 7F). Meanwhile, tumour weight was elevated after NEDD4L silencing and significantly reduced after KLF5 inhibition (Figure 7G). Overall, NEDD4L/KLF5 regulated ESCC radioresistance.

## 4 DISCUSSION

ESCC develops from precancerous lesions and its 5‐year survival rate is ~18%, a number reflecting its late diagnosis, aggressiveness and a lack of effective therapeutic schemes.^22^ Radiotherapy is among the primary clinical treatments for oesophageal cancer.^23^ Nevertheless, on the one hand, radiation‐induced DNA damage response can promote cytokine and chemokine production, provoke inflammatory reactions and tumour microenvironment changes, and thereby degrade the immune function and lead to ESCC invasion and metastasis.^4^ On the other hand, in ESCC, cancer stemness renders cancer cells to be extremely resistant to conventional therapies.^24^ Thus, combating radiotherapy resistance may be an indispensable part of ESCC treatment.

Cancer cells are susceptible to oxidative turbulence because of active metabolism and high ROS load, while oxidative stress via excess iron is linked with ferroptosis.^25^ As a key transcriptional regulator, KLF5 is normally expressed in oesophageal squamous epithelial cells and decreased or absent in human ESCC.^26^ Meanwhile, KLF5 is a potential transcription factor targeting ferroptosis regulators in cancer.^27^ Our results indicated that KLF5 downregulation increased lipid peroxidation, iron content, and ACSL4 and MDA expression and decreased SOD, GSH, and GPX4 levels, accompanied by morphological cellular changes that are characteristics of ferroptosis, indicating KLF5 knock down‐mediated promotion of ESCC ferroptosis. In addition, chronic exposure to ferroptosis‐inducing conditions may result in the accumulation of DNA damage.^28^ When DNA damage forms DSBs, it is followed by H2AX phosphorylation, and γH2AX is the first step in the recruitment and localization of DNA repair proteins.^29^ After KLF5 inhibition in ESCC cells, γH2AX nuclear translocation was enhanced. DNA‐PKcs and LIG4 are chemical inhibitors targeting key DNA damage response proteins and BMI1 ubiquitinates histone H2A and γH2AX thereby promotes the repair of DSBs.^30^, ^31^ RAD9 participates in promoting resistance to DNA damage and DNA repair.^32^ Our results exhibited that the levels of aforementioned genes participating in DNA repair were augmented to some extent after KLF5 downregulation. Collectively, KLF5 inhibition could exacerbate ROS accumulation and DNA damage in ESCC.

KLF5 undergoes different posttranslational modifications that modulate its protein level or transactivation activities such as phosphorylation and ubiquitination, and the former positively regulates its activity while the latter negatively modulates its protein level.^17^ NEDD4L has been reported to inhibit cell viability, cell cycle progression and glutamine metabolism in ESCC via ubiquitination of c‐Myc.^10^ Our results demonstrated that KLF5 protein levels were elevated with the decline of NEDD4L protein level and NEDD4L reduced KLF5 stability. Hence, it's inferred that NEDD4L interacted with KLF5 to diminish its stability by inducing ubiquitination.

Based on the above mechanism of function, we looked into the role of NEDD4L in ESCC ferroptosis and radiotherapy resistance. Increased NEDD4L in glioma cells induces accumulation of intracellular ROS levels, accompanied by decreased expression of key ferroptosis factors Nrl2 and GPX4.^33^ Consistent findings were obtained in our study, added that KLF5 inhibition averted the changes induced by NEDD4L knockdown. These findings elucidated that the NEDD4L/KLF5 axis has a regulatory function on ferroptosis of ESCC cells. NEDD4L induces OGG1 ubiquitination and affects cellular DNA damage response.^34^ Our results suggested that NEDD4L silencing decelerated DNA repair while KLF5 inhibition abolished the effect of NEDD4L silencing in ESCC. The possible mechanisms of radioresistance consist of cancer stem cells, DNA damage repair, ROS scavenging, epithelial–mesenchymal transition and programmed cell death.^4^ We further investigated the proliferation, invasion and migration of ESCC cells and observed tumour growth in vivo after modulating NEDD4L and KLF5. Silenced NEDD4L facilitated ESCC cell proliferation, migration and invasion and inhibited apoptosis in vitro, and accelerated tumour growth in vivo, while KLF5 inhibition produced an opposite effect. Taking these observations together with aforementioned effects of NEDD4L/KLF5 on ROS, ferroptosis and DNA damage, we thereby illustrated that NEDD4L/KLF5 could manipulate ESCC resistance to radiotherapy. According to our results, potential strategies to overcome the challenges posed by spatial heterogeneity, such as combination therapies or localized delivery methods, to enhance the targeting of the NEDD4L/KLF5 axis across different tumour regions. Moreover, diverse clonal populations within ESCC tumours and how this diversity might affect the sensitivity of tumour cells to NEDD4L/KLF5 axis inhibition. Certain clonal populations might exhibit resistance mechanisms that could undermine the overall therapeutic efficacy.

**FIGURE 8:**
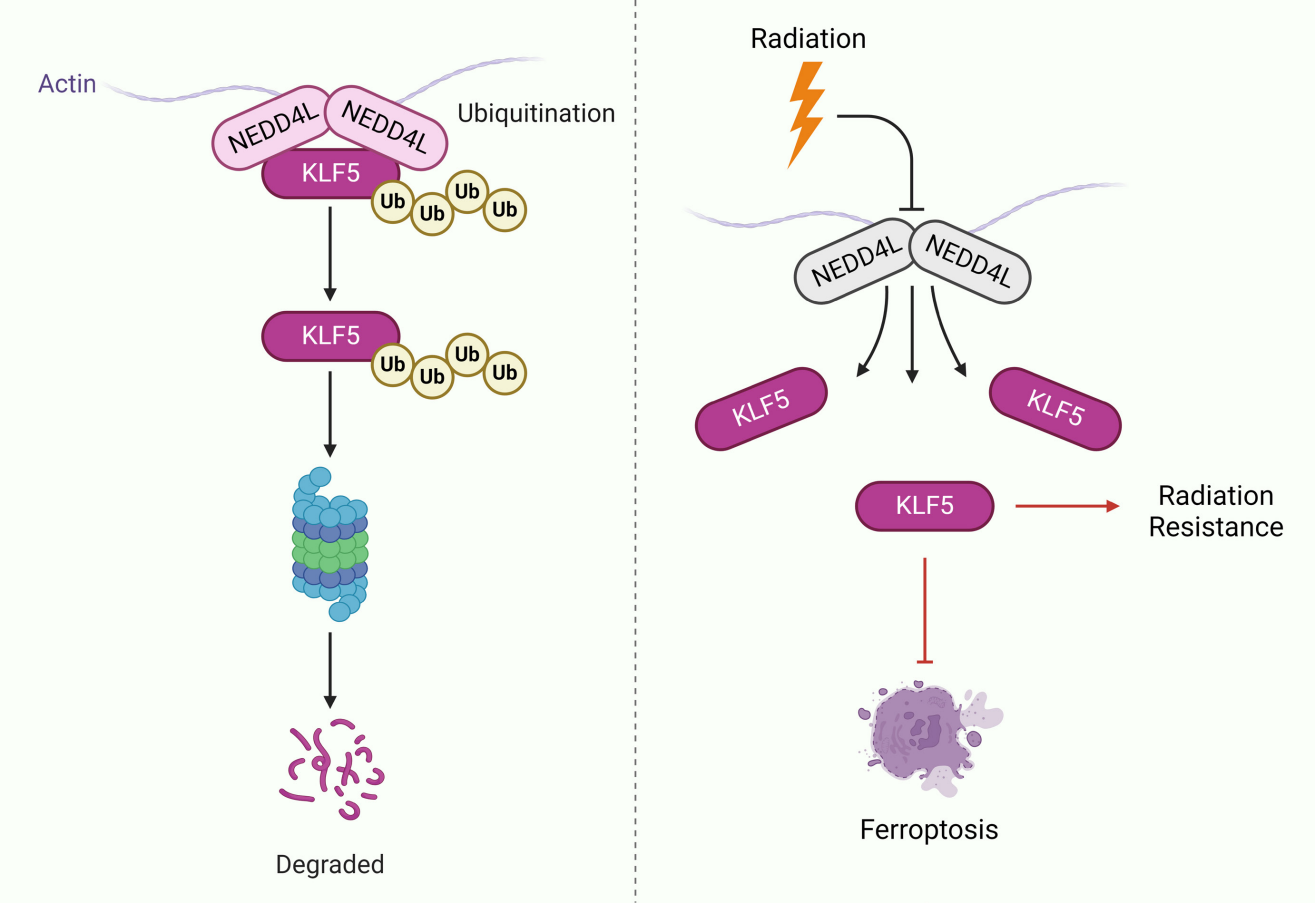
NEDD4L increases radiosensitivity by accelerating cellular ferroptosis via ubiquitination modification of KLF5.

In summary, our study results suggest that KLF5 can contribute to radiotherapy resistance in ESCC cells by reducing ROS‐induced ferroptosis. Additionally, KLF5 undergoes translational modification by NEDD4L, which promotes KLF5 ubiquitination, leading to protein instability and subsequent degradation (Figure 8). Although this study has several limitations such as the lack of detailed investigation of the upstream and downstream of NEDD4L and the pathways that may be involved in this mechanism, it has provided an insight into improving the effects of therapeutic strategies for ESCC. Still, further research is required for in‐depth study to better improve the prognosis of ESCC patients in the future. However, there still were several limitation of the study:

One limitation of this study is that it primarily focused on the effects of KLF5 inhibition on ROS accumulation, ferroptosis induction and radiosensitivity in ESCC cells induced by radiotherapy. While the results provide valuable insights into the role of KLF5 in these processes, the study primarily utilized in vitro cell culture models. Future studies should consider incorporating more complex models, such as in vivo models or patient‐derived samples, to better mimic the tumour microenvironment and validate the translational potential of these findings in clinical settings. Additionally, exploring the specific mechanisms underlying the interaction between KLF5, NEDD4L, and cellular responses to radiotherapy could provide further mechanistic insights.

## AUTHOR CONTRIBUTIONS

**Jinjin Chen:** Writing – original draft (equal). **Kaijun Ying:** Data curation (equal). **Jian Sun:** Resources (equal). **Yao Wang:** Formal analysis (equal). **Mingming Ji:** Validation (equal). **Yunhao Sun:** Writing – review and editing (equal).

## FUNDING INFORMATION

This study was supported by Scientific research project of Jiangsu Provincial Health Commission (Z2022062). The Basic research project of Yancheng Science and Technology Bureau (YCBK2023035).

## CONFLICT OF INTEREST STATEMENT

The authors of the research affirm that they had no commercial or financial relationships that could increase the likelihood of a conflict of interest.

## Data Availability

The data are available from the corresponding author on reasonable request.
